# Protocol for a randomized control trial of the caregiver support intervention with Syrian refugees in Lebanon

**DOI:** 10.1186/s13063-020-4175-9

**Published:** 2020-03-18

**Authors:** Kenneth E. Miller, Maguy Arnous, Fadila Tossyeh, Alexandra Chen, Ioannis Bakolis, Gabriela V. Koppenol-Gonzalez, Nayla Nahas, Mark J. D. Jordans

**Affiliations:** 1 Research and Development, War Child Holland, Amsterdam, The Netherlands; 2 War Child Holland Lebanon Offices (Beirut and Tripoli), Beirut and Tripoli, Lebanon; 3 Psychology Department, Harvard University, Cambridge, MA USA; 4 Institute of Psychiatry, Psychology, and Neuroscience at King’s College London, London, UK; 5 University of Balamand, Tripoli, Lebanon; 6 Amsterdam Institute of Social Science Research, University of Amsterdam, Amsterdam, Netherlands

**Keywords:** Refugees, Parenting, Mental health, Stress, Mindfulness, Syria, War, Distress, Children, Psychosocial

## Abstract

**Background:**

There is evidence that chronic stress negatively impacts parenting among refugees and other war-affected communities. Persistent parental stress and distress may lead to unresponsive, anxious, or overly harsh parenting and a corresponding increase in emotional and behavior problems among children. Most parenting interventions emphasize the acquisition of knowledge and skills; however, this overlooks the deleterious effects of chronic stress on parenting. The Caregiver Support Intervention (CSI) aims to strengthen quality of parenting skills by lowering stress and improving psychosocial wellbeing among refugee caregivers of children aged 3–12 years, while also increasing knowledge and skills related to positive parenting. The CSI is a nine-session psychosocial group intervention delivered by non-specialist providers. It is intended for all adult primary caregivers of children in high-adversity communities, rather than specifically targeting caregivers already showing signs of elevated distress.

**Methods/design:**

The primary objective of this study is to assess the effectiveness of the CSI through a parallel group randomized controlled study with Syrian refugee families in North Lebanon. Participants will be primary caregivers of children aged 3–12 years, with one index child per family. Families will be randomized to the CSI or a waitlist control group.

A total of 240 families (480 caregivers) will be recruited into the study. Randomization will be at the family level, and CSI groups will be held separately for women and men. The study will be implemented in two waves. Outcomes for both arms will be assessed at baseline, post-intervention, and at a 3-month follow-up. The primary outcome is quality of parenting skills. Secondary outcomes include parental warmth and sensitivity, harsh parenting, parenting knowledge, and child psychosocial wellbeing. Putative mediators of the CSI on parenting are caregiver stress, distress, psychosocial wellbeing, and stress management.

**Discussion:**

This trial may establish the CSI as an effective intervention for strengthening parenting in families living in settings of high adversity, particularly refugee communities.

**Trial registration:**

International Society for the Registration of Clinical Trials, ISRCTN22321773. Registered on 5 August 2019

## Background

Studies and field reports indicate that chronic adversity is generating high levels of stress among Syrian caregivers (in this paper, we use the term *caregivers* to refer to any primary caregivers of children, most but not all of whom are their biological parents). This includes those still in Syria as well as Syrian refugees in Lebanon and other adjacent countries [1–5]. In addition to coping with the impact of war-related experiences of violence and loss, Syrian refugee caregivers are contending with a host of ongoing stressors. These include poverty, inadequate and unsafe housing, severe restrictions on employment, a lack of access to healthcare, limited educational opportunities for their children, and the loss of social support networks [6–9].

Persistently high stress depletes caregivers’ coping resources and has been linked to compromised parenting, including unresponsive, overprotective, and harsh parenting. Compromised parenting, in turn, poses significant risks to children’s psychosocial and cognitive development [10–13]. For young children, it also represents a threat to the development of healthy attachments. Highly stressed and anxious caregivers are significantly more likely to have children with insecure attachments, which pose a risk for subsequent difficulties in children’s self-regulation, interpersonal relationships, and future academic achievement [12–15]. The linkages among parental stress, compromised parenting, and child mental health and psychosocial difficulties have been well-documented in studies of diverse refugee communities [6, 7, 16–19], including Syrian refugees in Lebanon [7, 20], the target population in the present study.

In response to growing evidence demonstrating the mediating role of parenting in shaping children’s responses to armed conflict and forced migration, non-governmental organizations (NGOs) have developed or adapted interventions aimed at strengthening parenting in refugee families. The primary emphasis in most such programs is on strengthening parenting knowledge and skills. This emphasis assumes, at least implicitly, that a deficit in parenting knowledge and skills underlies suboptimal parenting in highly stressed refugee families. An alternative hypothesis, supported by a growing number of studies, is that compromised parenting in refugee families reflects, at least partly, the impact of chronic adversity on the ability to parent effectively [6, 7, 18].

Parenting programs focused on knowledge and skills development *have* shown small to moderate effects on parenting outcomes in several studies, suggesting that addressing caregiver wellbeing directly may not be essential to achieving some benefit [21]. It remains to be seen whether the modest effects attained thus far might be strengthened by making stress reduction and improved caregiver wellbeing primary targets rather than collateral outcomes of parenting interventions.

**Fig. 1:**
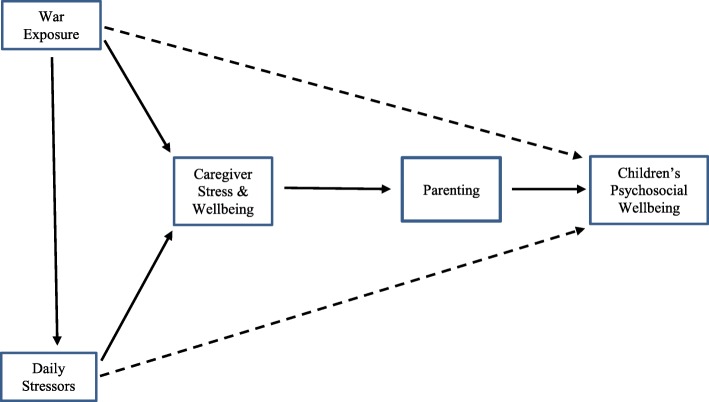
Conceptual model underlying the CSI

The Caregiver Support Intervention (CSI) has a dual focus on strengthening caregiver psychosocial wellbeing and increasing knowledge and skill regarding parenting under conditions of adversity. The guiding hypothesis underlying the CSI is that under conditions of lowered stress and increased wellbeing, caregivers will become better able to make more effective use of both pre-existing and newly acquired parenting knowledge and skills. Figure 1 depicts the model that guided the development of the CSI. The dotted lines show the relationship between war exposure and daily stressors with children’s wellbeing. The solid lines illustrate the key model underlying the CSI, in which war exposure and daily stressors adversely affect caregiver wellbeing, which in turn negatively affects parenting, ultimately impacting children’s wellbeing.

This paper presents the study protocol for a randomized controlled trial (RCT) of the CSI with Syrian refugee families in North Lebanon. A recently completed pilot RCT of the CSI in the same region and population with 79 families (151 caregivers) demonstrated the feasibility of all study methods [6]. Although we did not power that study to detect between-group differences, analysis of within-group differences between baseline and immediate post-intervention showed significant changes with medium or larger effect sizes in the expected direction on all parent-completed outcomes in the CSI group; in contrast, there were no significant changes on any outcome in the waitlist control group.

### Goals and objectives

The primary goal of this RCT is to evaluate the effectiveness of the CSI among Syrian refugee and host community caregivers in northern Lebanon.

#### Specific objectives

1. To assess the impact of the CSI on parenting
2. Secondarily, to assess the role of aspects of caregiver wellbeing (stress, distress, psychosocial wellbeing, and stress management) in mediating the impact of the CSI on parenting
3. Secondarily, to assess the impact of the CSI on parental warmth and sensitivity, harsh parenting, knowledge of positive parenting and child development, and child psychosocial wellbeing

## Methods/design

### Design

This will be a parallel group superiority RCT with an intent to treat design, a 1:1 allocation ratio, and a waitlist control comparison group. The study will be conducted in two waves, due to the large number of participants. Recruitment for each wave will take place in the neighborhoods of greater Tripoli (see the “Setting” section), with wave 2 recruitment starting during implementation of wave 1. This will help us ensure a high quality of implementation and rigorous data collection at all assessment points.

### Setting

The Government of Lebanon estimates that 1.5 million Syrian refugees, 54% of them children, are currently living in Lebanon, with about 1 million registered with the United Nations High Commissioner for Refugees (UNHCR) [22]. Sixty-nine percent of Syrian families in Lebanon live below the poverty line. This study will take place in Northern Lebanon, one of the most impoverished regions in the country. Specifically, the study will take place in the greater Tripoli/T5 region in the governorate of North Lebanon. As of January 2017, roughly 70,000 registered Syrian refugees were living in Tripoli; however, the actual number is widely understood to be much higher, as many Syrians have not registered as refugees with the UNHCR [22]. A 2017 study by the Feinstein Center found that approximately 75% of Syrians in Tripoli are living below the poverty line, with a monthly income below 180,000 LBP ($120). Their living conditions are generally precarious, work restrictions are increasingly stringent, and wages are among the lowest in Lebanon [8].

All assessments and intervention groups will be held in the community centers of community-based organizations (CBOs) with which War Child Holland collaborates, in or adjacent to the target communities. To be eligible to host the intervention, partner CBOs must have adequate space to conduct CSI groups with 12 adults, as well as a separate and adequately large space for childcare during the intervention and data collection.

### Participants

Participants will primarily be Syrian refugee caregivers of children aged 3–12 years, with one index child per family. Up to 25% of the sample may be comprised of Palestinian refugee and/or Lebanese families, in keeping with the Lebanon government’s policy of ensuring that programming for refugees is also available to host communities. In families with more than one child in the 3–12 year age range, the research coordinator will select one child randomly by rolling a die. In the Standard Operating Procedure document on the selection of the index child, a table specifies the meaning of the die roll. For example, if a family has two eligible children, a roll of one–three means the younger child is selected, while four–six means the older children is selected. Estimated total sample size is 240 families and 480 parents/caregivers (see the “Statistical power and sample size” section).

### Inclusion criteria

- Syrian refugee or vulnerable host community families with at least one child between the ages of 3–12 years
- Both primary caregivers are willing to participate in the study and willing to commit to attending all nine sessions of the CSI if randomized to the CSI arm of the study
- Participating caregivers are Arabic speaking

### Exclusion criteria

- Prior or current participation by either caregiver in a parenting or stress management intervention
- Family does not have a child aged 3–12 years
- Anyone who is unable, even with assistance, to complete the assessment questionnaires
- Unwillingness of either caregiver to give informed consent

### Intervention

The CSI is a nine-session weekly group intervention, co-facilitated by trained non-mental health professionals, who receive 6 days of training, three on-site observations with feedback, and weekly supervision. Groups are offered separately to women and men and are run with 10–12 participants. Table 1 lists session topics and corresponding modules, along with the stress management technique(s) taught in each session.

**Table 1:** CSI sessions, modules, and stress management/relaxation methods

Session	Topic	Module
1	Introduction and group building SM*: Participants’ own methods of coping with stress	Caregiver wellbeing
2	Stress and relaxation *SM: Counting the breath*	Caregiver wellbeing
3	Lowering our stress *SM: Stepping back from our thoughts, grounding*	Caregiver wellbeing
4	Coping with frustration and anger *SM: Peaceful walking, various anger management techniques*	Caregiver wellbeing
5	Parental stress and influence *SM: Stepping back from our thoughts (repeat)*	Parenting in adversity
6	Increasing our influence as parents, part I: Positive attention *SM: Guided visualization: A safe place*	Parenting in adversity
7	Increasing our influence as parents, part 2: Effective discipline *SM: Informal breathing practice*	Parenting in adversity
8	Positive parenting: Practice *SM: Participants choose any stress management/relaxation method*	Parenting in adversity
9	Looking back, looking forward	Closure

*SM* stress management/relaxation technique taught during the session

Sessions 1–4 are focused on strengthening caregiver wellbeing, with individual sessions on understanding and managing stress, disengaging from “thinking too much”—a culturally salient phenomenon that both indicates and exacerbates stress, roughly akin to rumination [23], and coping with anger and frustration, all while developing the group as a socially supportive setting. Sessions 5–8 focus on strengthening parenting under conditions of adversity (i.e., increasing awareness of the impact of stress on parenting, increasing positive parent–child interactions and the use of non-violent discipline methods, and reducing harsh parenting). Session 9 involves a review and closing of the intervention. In all but the final session, participants learn a new relaxation or stress management technique, drawn or adapted from the mindfulness and stress management practice. These techniques are also provided to participants in Arabic on mp3 files, which they can listen to on their smart phones or on mp3 players provided at the start of the program. Participants are encouraged to practice these activities at least three times each week. A considerable amount of time is spent at the start of each session reviewing the home practice and collectively problem-solving any barriers to practicing the techniques.

### Control group

This study is employing a waitlist control as the comparison group, based on our successful use of the same design in the pilot RCT of the CSI. Participants in the waitlist control group in each wave of the study will be invited to participate in CSI groups that will begin shortly after the 3-month follow-up assessment has been completed.

### Recruitment

Participant recruitment will be conducted in collaboration with the staff of the local CBOs with which War Child has collaborative relationships in each target community. Community breakfasts to announce the study, door to door recruitment—especially important for recruiting men—along with visits by research staff to settings where men commonly gather, flyers posted in local partner CBO offices, and word of mouth will all be used to recruit participants into the study. In order to recruit men/male caregivers, we will utilize the same strategies that proved effective in the pilot RCT: scheduling assessments and intervention sessions on days and at times that do not conflict with income-generation opportunities, training our recruitment team on the importance of men’s participation in the CSI, and crafting a recruitment message that emphasizes both the stress management and parenting foci of the intervention (Miller KE, Koppenol-Gonzalez GV, Arnous M, Tossyeh F, Chen A, Nahas N, et al.: Supporting families displaced by armed conflict: A pilot randomized controlled trial of the caregiver support intervention, submitted).

### Measures

All questionnaire data will be gathered using tablets, using the software program Kobo, which allows questionnaires to be completed and uploaded without paper and pencil. Kobo is available free of charge from the Harvard Humanitarian Initiative (https://www.kobotoolbox.org/). Measures will be administered in Arabic to each parent/caregiver individually by trained and supervised research assistants. See Table 2 for an overview of all measures.

**Table 2:** Measures

Domain/construct	Tool	Items
*Primary outcome*		
Parenting	Subscale of new parenting measure	24
*Secondary outcomes*		
Parental warmth and sensitivity	Subscale of new parenting measure	14
Harsh parenting	Subscale of new parenting measure	5
Parenting knowledge	Subscale of new parenting measure	15
Child psychosocial wellbeing	Kid and Kiddy KIndl-Parent report	24
*Mediators*		
Caregiver distress	K10	10
Caregiver stress	New measure developed for this study	8
Caregiver psychosocial wellbeing	Warwick Edinburgh Mental Wellbeing Scale	14
Caregiver stress management	New measure developed for this study	10
*Exploratory outcome*		
Infant/toddler mental health and socioemotional development	CREDI*	20

* The CREDI will be used with families with a child aged 0–3 years

Three new measures were developed for this study: Caregiver stress, Stress management, and Parenting. The development and piloting of these measures is described in a forthcoming paper. Briefly, we decided to develop new measures for these key outcomes after we searched the literature extensively and were unable to identify measures that [1] were suitable for caregivers with children of such a broad age range (3–12 years); had been validated for use with Syrians, Lebanese, or Palestinians; and [3] were worded in ways that would be deemed acceptable in the target communities. All items on the new questionnaires were drafted in English (with Arabic terminology and idioms in mind), reviewed by a panel of experts, translated into Arabic and back-translated into English, with all discrepancies resolved through a consensus process among bilingual project staff. The items were then assessed for ease of understanding and cultural acceptability through a process of cognitive interviewing with groups of Syrian caregivers from the target community. All items were deemed acceptable and were readily understood. Minor wording changes were made to several items to ensure the intended meaning was conveyed. This same process of cognitive interviewing was also undertaken with the other questionnaires in the study. All measures, including the three newly developed questionnaires, were then administered to a group of 50 caregivers on two occasions, one week apart, to assess their internal consistency and test–retest reliability. The internal consistency and test–retest reliability of the three new measures ranged from acceptable to high, as described below.

#### Parenting

Parenting will be assessed using a new 24-item Parenting Scale for this study. In addition to yielding a total score, the measure includes subscales assessing *parental warmth and sensitivity* (14 items) and *harsh parenting* (five items). Internal consistency for the full measure in our pilot study was good (α = 0.87), as it was for the warmth and harsh parenting subscales (α = 0.84 and α = 0.76, respectively). Test–retest reliability of the full scale was acceptable (ICC = 0.67, 95% CI 0.49–0.79); it was good for the parental warmth and sensitivity subscale (intra-class correlation coefficient (ICC) = 0.77, 95% confidence interval (CI) 0.62–0.86), and acceptable for the harsh parenting subscale (ICC = 0.69, 95% CI 0.51–81). Using baseline data from the sample in this study, we will examine the factor structure of the Parenting measure to see whether it fits our hypothesized structure. The Parenting measure also includes a separate 15-item Parenting knowledge scale assessing knowledge of positive parenting methods and early childhood development. It is scored separately from the other items on the parenting questionnaire, using a simple true/false answer choice option.

#### Caregiver stress

Caregiver stress will be assessed with an eight-item scale developed for this study. In our pilot RCT, the scale showed good internal consistency (α = 0.75). In our measure development study, it demonstrated good test–retest reliability (ICC = 0.86, 95% CI 0.75–0.92).

#### Caregiver psychological distress

The Kessler Psychological Distress Scale [24] is a widely used ten-item measure of psychological distress. It has been used extensively in cross-cultural clinical and epidemiological research, and has demonstrated excellent psychometrics in diverse populations. It showed a high level of internal consistency (alpha = 0.88) and convergent validity in a recent study of adults in Palestine [25]. The internal consistency of the K10 in our pilot RCT was good (α = 0.86), and test–retest reliability was acceptable (ICC = 0.74, 95% CI 0.59–0.85).

#### Stress management

Stress management was assessed using a ten-item scale developed for this study. Internal consistency was good (α = 0.76), and test–retest reliability in our measure development study was acceptable (ICC = 0.72, 95% CI 0.52–0.84).

#### Caregiver psychosocial wellbeing

The Warwick-Edinburgh Mental Wellbeing Scale [26, 27] is a 14-item measure of psychosocial wellbeing that has been used extensively in cross-cultural mental health research, and has demonstrated good psychometrics in diverse populations. The internal consistency of the WEMWBS in our pilot RCT was good (α = 0.72). Test–retest reliability was good (ICC = 0.78, 95% CI 0.61–0.88).

#### Child psychosocial wellbeing-parent report

Children’s psychosocial wellbeing will be assessed with the Kid-KINDL for Parents [28] for index children aged 7 years and older, and the Kiddy-KINDL for Parents for children aged 3–6 years. The four school items were dropped to make the two versions identical, and four optional mental health items were added to strengthen our measure of their psychosocial health, yielding a total of 24 items. Because mothers spend considerably more time with their children in Arab culture, only female caregivers will be asked to complete the Kindl. Internal consistency of the parent-completed Kindl in our pilot study was good (α = 0.83), and test–retest reliability in our measure development study was acceptable (ICC = 0.76, 95% CI 0.59–0.86). During our pilot study, we also used a child-self report version of the Kid-Kindl; however, due to its marginally acceptable test–retest reliability, the week correlation with the parent-report version, and the fact that only a subset of index children was deemed old enough to complete the measure (ages 7–12), we have decided in the present study to gather data only from caregivers.

#### Demographics

A brief demographics form, developed for the pilot RCT, will be used to record family composition, caregiver nationality, sex, and age, ages and sex of all members of the household, years in Lebanon (if non-Lebanese), and other demographic variables relevant to the study’s outcomes.

### Informed consent

Informed consent will be gathered at the baseline assessment, prior to gathering any data. The research coordinator will distribute a consent form in Arabic, and read it aloud to participants to ensure full comprehension regardless of their literacy levels. The research coordinator will allow time to address all questions and concerns participants may raise.

### Randomization and blinding

After caregivers have completed the baseline assessment, families will be randomized to the CSI or a waitlist control group. Randomization will be at the family level, to ensure that caregivers from the same family are not randomized to different arms of the study; 240 families will be randomized to the CSI or control arm. CSI groups will be held separately for women and men, yielding a total of 120 families and 240 caregivers per arm.

As we are running the study in several communities, a block randomization design will be used, using a participatory methodology implemented successfully in our pilot RCT, and adapted from the work of Mercy Corps and Yale University in their study of a life skills program for Syrian refugee children in Jordan [29]. At baseline assessment, after completing the questionnaires, one caregiver from each family will be asked by a research team staff member to draw a lollipop out of an opaque bag filled with an equal number of red and green lollipops to ensure an equal number of CSI and waitlist control participants. Caregivers will be told that after baseline data have been completed, a coin toss will determine the meaning of each color: one color will mean CSI and the other color will mean waitlist control. This process will be repeated in each of the communities where the study will be conducted, leading to an equal number of CSI and WLC families in each community and in the study as a whole. After the coin toss, done by a staff member of War Child Holland unaffiliated with the study, the outreach team will inform all participants of their group assignment and let CSI participants know the day and time of their weekly group sessions. A research team member will manage the lollipop selection, while a War Child staff member unaffiliated with the study will toss the coin.

The purpose of this two-step randomization process is to increase community buy-in to the randomization process, by demystifying it and giving participants an active role in the process. We successfully randomized participants in the pilot study in this way (there were no significant between-group differences on any variable following randomization). Moreover, participants understood the process and expressed a willingness to accept assignment to either the CSI or waitlist control arm. This willingness was confirmed by the high percentage of WLC participants who completed the post-intervention assessment (99%) in the pilot study.

A master list will be created that includes each family’s group assignment. This list will be kept in a secure location in the War Child Holland Tripoli office, with a copy in a similarly secure location in the War Child Office in Beirut. Only the research coordinator and the research implementation coordinator will have access to this master file during the study.

Given the nature of the study, participants and facilitators will not be blinded to group assignment. Research assistants (RAs) completing the baseline and follow-up assessments *will* be blind to group assignment and therefore will not be involved in the randomization process. RAs will be instructed never to ask any participant to reveal their group assignment, and to gently stop participants from revealing their group assignment if they begin to do so during the post-intervention assessments. As we did in the pilot study, we will also explain to participants at the post-intervention assessments the importance of not revealing their group assignment to the RA during the assessment. There is no planned contact between the RAs and CSI facilitators, and we will instruct RAs to never discuss any research participant outside of the data collection process, except with the research coordinator, both for confidentiality and to ensure against any possible breach of blinding to participant assignment. The research coordinator and CSI trainer/supervisor will not be blind to group assignment, as they will be involved in scheduling participants into specific groups. The principal investigator (PI), co-PIs, and trial statisticians will all be blind to group assignment.

### Baseline, post-intervention, and follow-up assessments

**Fig. 2:**
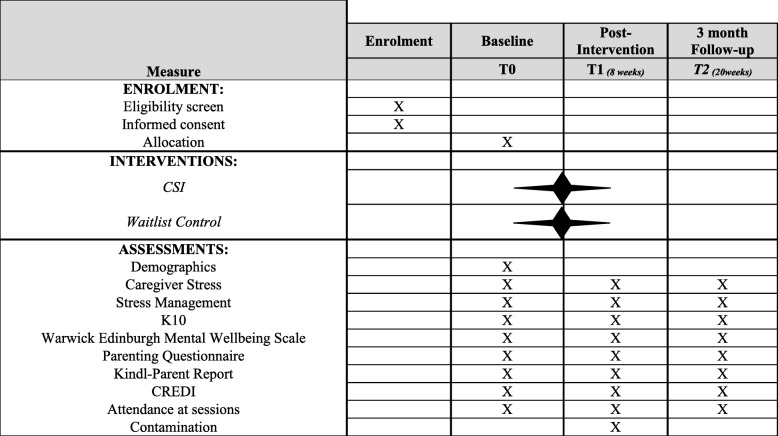
Standard Protocol Items Recommendations for Interventional Trials (SPIRIT): Schedule of enrolment, interventions, and assessments for trial of CSI

Questionnaire data will be gathered at three time points: baseline, post-intervention, and 3-month follow-up. Because the study will be implemented in two waves, there will be a total of six assessments, three in each wave. This is depicted in the SPIRIT figure chart in Fig. 2.

**Fig. 3:**
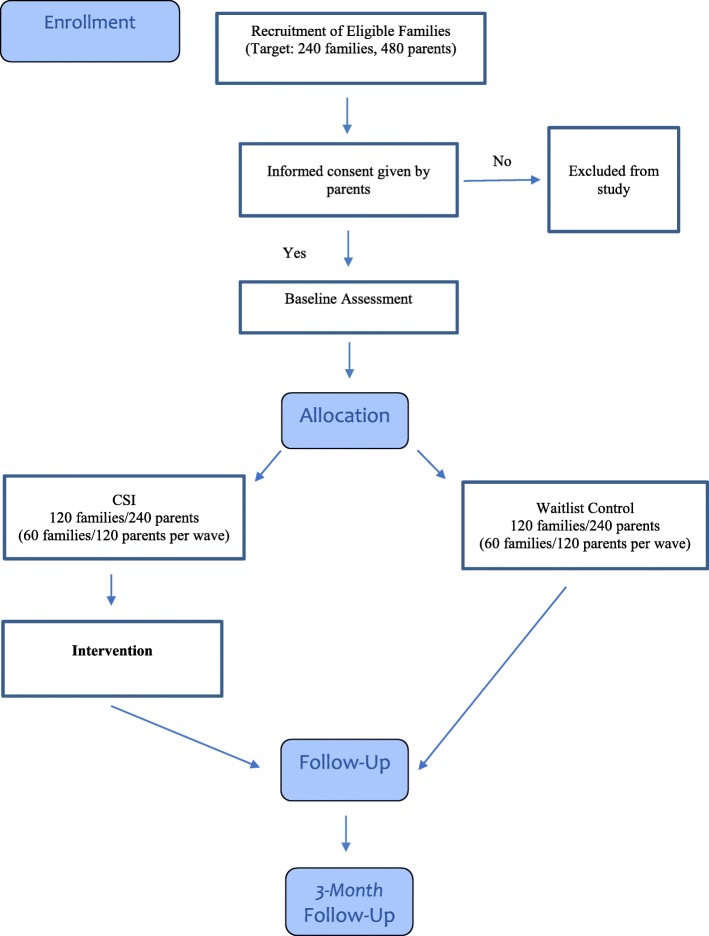
Study flow chart

Participant flow, from enrollment through the 3-month follow-up assessment, can be seen in Fig. 3.

### Retention of participants in the study following post-intervention assessment

In the pilot study, retention of the WLC group was facilitated by periodic WhatsApp text messages reminding WLC participants of the post-intervention assessment and the CSI intervention that would follow shortly thereafter. In the present study, there will be an additional three and a half month period due to the follow-up assessment before the WLC group receives the intervention. In order to maintain interest in the study, a separate social event will be held for the WLC and CSI groups in each wave. The event will be a gathering in a park or community center with food and music for a few hours, and will occur during the month following the post-intervention assessment.

### Statistical power and sample size

A target of 480 caregivers (240 families) will be recruited in two waves. We initially calculated a sample size based on 90% power at 5% level of significance and an attrition rate of 20%, yielding a sample of 432 individuals or 216 families. However, because we aim to have 12 participants in each CSI group, we increased the sample size to 480 (240 families). Dividing the sample over two waves, with randomization into CSI and waitlist control in each wave, this sample size yields a total of ten CSI groups per wave (five for women and five for men), for a total of 20 CSI groups.

Target population and parameters: The primary target population is Syrian refugees living in northern Lebanon, with secondary populations being Palestinian refugee and Lebanese families in the same or adjacent communities, who have at least one child 3–12 years old. In our pilot study of the CSI, the baseline total score of our newly developed parenting scale was 61.6. This is a new scale so there are no direct comparisons from the literature about the expected mean total score of this measure. The mean total score of parenting following the CSI intervention was 63.7 at 9 weeks. As our primary outcome is a continuous variable, it can be described by the normal distribution. The final analysis will incorporate baseline mean rate as a covariate to improve the precision of the estimate of the treatment difference between groups. In order to estimate the within-group correlation between baseline and outcome rate (of total parenting score) we ran a Pearson’s correlation between baseline and 9-week scores in the CSI pilot study, giving a point estimate of ρ = 0.6. The intra-class correlation has been estimated at 0.15 based on a random intercept regression model with total parenting score as the outcome, CSI group as the intervention, and adjusting for baseline total parenting score. The STATA command *clustersampsi* was used for the power and sample calculation.

#### Data analysis

The statistical analyses will be carried out by the trial statistician, who will remain blind to the group randomization until the main analyses are complete. The analyses outlined in this strategy will be primarily based on an intention to treat (ITT); a per protocol (PP) analysis will be the secondary analysis. The PP analysis will include participants who complete the CSI, with completion defined as attending at least seven of the nine sessions, and will exclude any participant who attended fewer than seven sessions. We have powered the study (see the “Sample size” section) as a superiority study at 10 weeks. The first stage of analyses will be a descriptive model of the data to assess completeness of data and the integrity of the data collection system. Participants and area characteristics and demographics will be summarized at baseline. Clinical characteristics that have been measured repeatedly will be summarized at baseline and at the post-randomization follow-up assessments. In addition, patterns of missing data will be described.

The primary outcome (total score of parenting scale) will be analyzed using linear mixed models to model the mean difference in the total score of parenting scale 10 weeks post-randomization. The linear mixed models will be adjusted for baseline total score of parenting scale and stratification. A two-level hierarchical model will be employed to improve power and take into account clustering of the parents at the family level.

These models utilize maximum likelihood estimation and thus allow for missing outcome data under the missing at random (MAR) assumption. Associations between post-randomization variables and missingness will be dealt with by multiple imputation (MI), again under the MAR assumption. Departures from this assumption will be assessed with a sensitivity analysis.

Secondary outcomes, including parental warmth and responsiveness, harsh parenting, parenting knowledge, caregiver psychosocial wellbeing, and child psychosocial wellbeing will be assessed with a similar methodology used for the primary outcomes, using generalized linear mixed models depending on the type of outcome (normal, ordinal).

### Evaluation of mediation

**Fig. 4:**
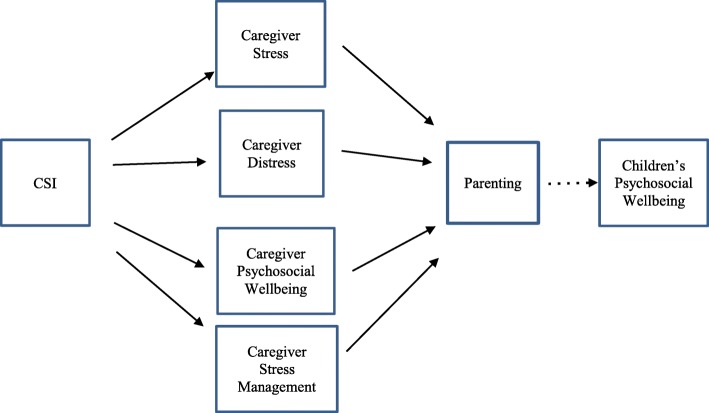
Putative mediation of impact of CSI on parenting by caregiver stress, caregiver distress, caregiver psychosocial wellbeing, and stress management. Solid lines represent mediational pathways to be tested

We will investigate the mediation process of different aspects of caregiver wellbeing and, through that, illuminate key basic knowledge about generalization of acquired skills in parenting through the CSI intervention. Some of the pathways of interest are illustrated in Fig. 4. If the efficacy analysis shows significant between-group differences in the mediators (caregiver stress (Caregiver Stress Scale), caregiver distress (K10), caregiver psychosocial wellbeing (WEMWBS), stress management), then we will use parametric regression models to:

- Test for mediation of the intervention on Parenting through caregiver stress (Caregiver Stress Scale)
- Test for mediation of the intervention on Parenting through caregiver distress (K10)
- Test for mediation of the intervention on Parenting through caregiver psychosocial wellbeing (WEMWBS)
- Test for mediation of the intervention on Parenting through stress management (Stress Management Scale).

Since all the measures are continuous, the indirect effects will be calculated by multiplying relevant pathways and bootstrapping will be used to produce valid standard errors for the indirect effects. All analyses will adjust for baseline measures of the mediators (Caregiver Stress Scale, caregiver distress (K10), stress management, outcome (Parenting scale) and putative measured confounders (e.g., socioeconomic status).

### Facilitators

Prospective CSI facilitators must meet the following criteria to be accepted into the 6-day training:

1. Native Arabic speaker
2. Preferably from the geographic locations of implementation
3. Minimum age 24 years
4. High school education required, university education desirable
5. At least 2 years of experience implementing psychosocial interventions, preferably with adults, even more preferably with parents/caregivers
6. Emotionally mature and receptive to the core ideas of the CSI regarding parental wellbeing and positive parenting, as assessed during an interview with the CSI lead trainer and supervisor and possibly other relevant WCH staff
7. Able to commit to attend the full training, all sessions of the intervention, and all supervision meetings
8. Respectful and tolerant to different nationalities and religious groups
9. Being a parent is highly desirable but not required
  - While we recognize the potential bias of being a parent (facilitators with children may bring in their own experiences and attitudes to the intervention), in our experience we have found that participants view facilitators with children as having greater legitimacy to lead sessions on parenting knowledge and skills; in addition, facilitator-parents may have a deeper appreciation for the challenges and stressors that parents face. We make every effort, in training, supervision, and on-site coaching, to ensure that any bias introduced to the sessions by facilitators, whether due to having children or any other source, is minimized.

### Facilitator training

CSI facilitators first participate in a 6-day training which combines didactic and experiential components, and includes extensive practice implementing the intervention within the training group. In addition to covering the specific content of the sessions (e.g., parental stress and wellbeing, relaxation exercises, coping with anger and frustration, child development, positive parenting), trainees also learn group facilitation skills through extensive role-playing and continuous feedback. The trainers complete a detailed competency checklist for each trainee at the start and completion of the training; trainees also complete a knowledge and attitudes assessment at the start and completion of the training. Only those trainees who are deemed sufficiently knowledgeable and skilled, based on a trainer-completed competency checklist and the pre-post knowledge assessment, are then invited to become CSI facilitators. During their first full implementation of the CSI, facilitators are observed a minimum of three times with the competency checklist completed on each occasion, and receive weekly supervision.

### Childcare during CSI sessions

Childcare will be provided on-site for up to two children per participant. Trained and experienced animators (child care providers) trained by War Child Holland in child care and child safety will care for children, with two animators available during each session. The childcare sessions are not structured, and no formal activities are facilitated, in order to avoid providing any index children attending the childcare with a substantive psychosocial experience that might confound the results of the study. Various play materials and snacks will be provided, and the child care workers will ensure the safety and comfort of all children.

### Intervention fidelity assessment, quality control, and supervision

A fidelity checklist will be completed jointly by the co-facilitators immediately after each session of the CSI. The number of items on the fidelity checklist varies by session, as it covers all of the activities specific to each session. The research implementation coordinator and the CSI trainer and supervisor will conduct three on-site observations in each group and provide coaching to all new facilitators based on these observations. They will use the fidelity checklist and a supplementary competency checklist to guide their feedback. They will also meet with all pairs of facilitators weekly for supervision. Registers will be used to record attendance at all sessions of all CSI groups. These will be kept in the secure care of the research implementation coordinator. The research implementation coordinator and CSI trainer and supervisor will receive their own supervision weekly from War Child Holland’s Lebanon-based psychosocial coordinator, and biweekly supervision from a psychologist and faculty member the University of Balamand (Nahas).

The PI will hold weekly meetings with the research coordinator and research implementation coordinator to review all activities and issues and ensure fidelity to the research protocol.

### Minimization of contamination

To minimize contamination, randomization will be at the family rather than individual parent level, in order to avoid having parents/caregivers within the same family assigned to different arms of the study. To minimize contagion between the CSI and control arms, participants in CSI groups will be asked to avoid sharing program content, including the relaxation and stress management exercises, with anyone outside of their immediate household.

### Masking of outcome assessments

All assessments will be carried out by trained research assistants (see the “Research assistants” section for details about background and training of the research assistants). Efforts will be made to minimize interactions between the intervention and research teams during the study. As noted earlier, the rationale for keeping the RAs blind is explained to participants at each assessment, along with a request that they not reveal their group assignment. This was accomplished successfully during the pilot study, and we anticipate no difficulties replicating this in the present study.

### Nested qualitative study

Following the intervention and within 3 weeks after the post-intervention data collection, focus groups discussions will be conducted by the research coordinator and a trained research assistant with all groups of CSI participants (male and female separately), to examine perceptions of the intervention, perceived impact on personal wellbeing and parenting, and barriers to accessing the intervention and to utilizing intervention content. The focus groups will also explore participants’ experience of completing the assessment battery (e.g., level of difficulty, perceived burden).

### Data management

Participants will be assigned a study ID, and their names will not appear on any data collected. Questionnaire data will be uploaded directly by the research coordinator to the secure KOBO server, from which it will be downloaded to WCH’s own secure server and entered into SPSS (quantitative) or NVivo (qualitative). All data will be backed-up securely at the WCH Amsterdam office, and all data and analysis files will be securely password protected and encrypted. For all questionnaire data, range and consistency checks will be performed within days post data-entry, which will occur immediately following each wave of data collection. Any queries identified will be resolved promptly by the trial management team. A detailed WCH data management policy will serve as guidance on all data management issues. Focus group notes will be taken verbatim in Arabic, translated into English, and entered into NVivo, where they will be coded and analyzed by the research team.

### Trial management

A committee will monitor the progress of the trial. Trial monitoring will comprise the collation and reporting of routine trial process indicators and adverse events (AEs)*.* Summary statistics and graphs showing trends over time will be compiled for the process indicators. No interim analyses are planned. In addition to the PI (Dr. Miller), and Dr. Mark Tomlinson, an international leader in the field of family and early childhood research, additional members of the trial management committee include Dr. Mark Jordans, co-investigator on the project and director of Research and Development at War Child Holland, and Maguy Arnous, the research coordinator for this study.

### Risk mitigation

The CSI is a non-pharmacological, non-clinical intervention. Although the target population is in a high-adversity setting, there is minimal risk to participant safety or wellbeing from participation in this study. During three earlier cycles of implementation including the pilot RCT, while the intervention was being developed and finalized, there were no instances of serious AEs, or of any AEs related to the intervention or research methods. Referrals *were* made for social or medical assistance in eight cases (illness, eviction, and gender-based violence). All CSI facilitators as well as all research assistants are trained to identify signs of distress and to report any possible AEs to their supervisor. All facilitators receive weekly supervision, both to ensure fidelity and quality of implementation and to support their own skill development and wellbeing.

### Adverse events

We will monitor the occurrence of AEs using the War Child Holland (WCH) Adverse Events Reporting Procedure. A data safety and management committee (DSMC) will provide oversight on AEs and safety protocols for the study. The DSMC will be composed of individuals to be named prior to the start of the trial, none of whom will be a part of this study. The key terms of reference for the DSMC will be to review reports of AEs (within 48 h of notification). Reviews should occur on a 12-monthly basis via Skype. The purpose of the DSMC is to monitor the occurrence of AEs and where required make decisions on further actions to be taken to determine whether AEs are likely to be related or unrelated to the intervention. The DSMC will have the mandate to recommend stopping the study if it is determined that the risks of the study outweigh the benefits.

All AEs reported spontaneously by participants or observed by the investigators or other staff members will be recorded by the research team on WCH’s Adverse Event reporting form. AEs can be detected by anyone as all study staff will be trained in their detection and management. Intervention facilitators will initially discuss AEs reporting with their supervisor, who will report this to the research coordinator. Research assistants will initially discuss AE reporting with the research coordinator. The research coordinator will subsequently share all AEs with the PI, who will be responsible for ensuring appropriate responses to all AEs. All AEs will be reported to the DSMC. This will occur within 24 h for AEs. The chair or nominated person from the DSMC will review AEs within 48 h and the DSMC will review all AEs monthly and where necessary to determine whether AEs are likely to be related or unrelated to the intervention.

### Criteria for discontinuing the intervention for study participants

Any participant may elect to discontinue their participation in the study at any point for any reason, including participation in the intervention. Any participant who becomes repeatedly disruptive to the intervention may be asked to discontinue participation in the intervention, but not the study.

### Compensation for participation/defraying costs of participation

A small reimbursement (approximately 5 USD) of transportation costs of caregivers and children to the organization offices and/or community centers will be provided for assessment sessions. Transportation will be provided for attendance at assessment and intervention sessions. Refreshments will also be provided at both intervention and assessment sessions. This compensation is considered minimal and in line with regular WCH implementation programming, and was chosen in order to avoid difficulties of jealousy within the community amongst non-eligible families, and potential coercion of families to participate in sessions and assessments.

### Research assistants

A team of 12 research assistants will be trained for a period of at least 5 days prior to the baseline assessment of wave 1. This group will include a sub-group of experienced research assistants who worked on the CSI pilot RCT, as well as newly trained RAs. Research assistants are native Arabic speakers, preferably with experience serving refugee and vulnerable communities, and will be recruited in Lebanon based on recommendations from local colleagues. Training will cover research ethics, child safeguarding, handling adverse events, building rapport with participants, communication with children, troubleshooting, and the use of the tablets, among other topics, and will include extensive practice administering all questionnaires to be used in the study.

### Ethical considerations

This research protocol has been approved by an institutional review board at the University of Balamand in Tripoli, Lebanon. Written (or witnessed, if the participant is not literate) informed consent by caregivers will be mandatory for enrollment in all research activities. We will protect the confidentiality of personal data principally through procedures to separate study data and participant identifiable data. No children will be involved as participants in this study.

### Dissemination of results and publication policy

The results of this project will be published in English in peer-reviewed journals (we will aim to publish in open access journals). They will be disseminated in Arabic and English to key stakeholders through reports and presentations. If the CSI is shown to be effective, an adaptation manual will be developed in the final months of the grant period to facilitate the implementation by War Child in other countries where the organization is active, and by other institutions and non-governmental organizations interested in using the CSI in their programming.

### Protocol amendments

Any substantive amendments to this trial protocol will be submitted to the institutional review board committee at the University of Balamand that approved the original protocol. Such amendments will also be submitted to ISRCTN for inclusion in the registered protocol.

## Discussion

The CSI was developed with the aim of strengthening parenting in refugee communities by [1] lowering the stress and increasing the psychosocial wellbeing of refugee parents, and [2] strengthening their parenting knowledge and skills. The ultimate aim is to create an evidence-based, scalable intervention that can be adapted and implemented in diverse refugee communities and contexts. The emphasis in the CSI on strengthening parental wellbeing as an approach to improving parenting represents a shift from conventional parent training programs, which prioritize the acquisition of knowledge and skills. If the findings of this study support the model underlying the CSI, this will lend support to the idea that parenting in settings of adversity can be strengthened by addressing parents’ own wellbeing as well as by increasing and their knowledge and skills.

A second contribution of this study, if our methods are effective, will be to demonstrate the feasibility of recruiting and retaining men/male caregivers in a parenting intervention. Panter-Brick et al*.* [30] have cautioned that the paucity of men in parenting interventions may be undermining the effectiveness of such programs, both because men may not support the changes their partners are trying to make at home and because men may continue to engage in problematic parenting behaviors themselves. In our pilot RCT of the CSI (Miller KE, Koppenol-Gonzalez GV, Arnous M, Tossyeh F, Chen A, Nahas N, et al.: Supporting families displaced by armed conflict: A pilot randomized controlled trial of the caregiver support intervention, submitted), we successfully recruited both caregivers in our target of 72 families, of whom 86% completed at least seven of the nine sessions. If we are able to successfully recruit 240 families in which both caregivers participate, we believe this may challenge the widespread perception that men are uninterested and unwilling to participate in parent-focused interventions.

Finally, we acknowledge the trade-off in deciding to focus exclusively on families in which both caregivers are willing to participate in the study. If this trial establishes the effectiveness of the CSI in families with both caregivers participating, it will be unclear to what extent we can expect to achieve similar effects in families with only one participating caregiver.

## Trial status

Enrollment for wave 1 of this study started on 25 September, 2019. Enrollment will end on or about 19 January, 2020.

## Data Availability

Data and materials will be shared upon request to Kenneth E. Miller, War Child Holland.
